# The (un-)Social Brain in Isolation

**DOI:** 10.33549/physiolres.935565

**Published:** 2025-10-01

**Authors:** Allen FISHMAN, Peleg GRININ, Vladimír RILJAK

**Affiliations:** 1 Institute of Physiology of the First Faculty of Medicine, Charles University, Prague, Czech Republic

**Keywords:** Chronic Stress, Loneliness, Social Cognition, Socialization, Social Stress

## Abstract

The Social Brain is a distributed network of neuroanatomical regions and neurochemical systems that underpins the human capacity for social cognition, empathy, and interpersonal behavior. Social isolation (SI), defined as the objective reduction in social interaction, poses a significant threat to the integrity of this system. In this review, we synthesize evidence from human and animal studies to elucidate the biological, cognitive, and behavioral consequences of SI on the social brain. We describe how SI acts as a chronic stressor, disrupting structural connectivity, and altering neurotransmitter systems critical for social cognition. These disruptions manifest in altered social behavior, mentalization processes, and emotional reactivity, significantly contributing to increased vulnerability to psychiatric and neurodegenerative disorders, including depression, schizophrenia, substance use disorders, and Alzheimer’s disease. Converging findings from studies of evolutionarily conserved mechanisms in rodent and primate models demonstrate that SI compromises neurodevelopment, attenuates neuroplasticity, and triggers maladaptive stress responses, highlighting that social deprivation has profound neurobiological and behavioral consequences that greatly overlap with the pathophysiological changes seen in neuropsychiatric disorders. Furthermore, we explore the role of indirect stressors resulting from SI such as touch deprivation and digital-era social disconnection as contemporary amplifiers of SI’s neurobiological impact. In light of public health challenges such as the COVID-19 pandemic, we propose that SI should be recognized not only as a psychosocial condition but as a modifiable risk factor with transdiagnostic significance across psychiatry, neurology, and preventive medicine. Addressing SI through targeted interventions and policy measures is essential for promoting mental resilience and well-being.

## Introduction

In this paper, we review the various direct (e.g. decreased number and/or frequency of social interactions, decreased physical contact) and indirect (e.g. chronic stress, altered neural processes, hormonal changes, alterations in gene expression) effects of isolation on the social brain’s biological processes and the resulting behavioral changes.

The Social Brain refers to a distributed network of brain regions and neurochemical systems responsible for processing, interpreting, and responding to social stimuli. This integrative system is central to a wide range of cognitive and emotional functions, including social perception, empathy, emotional regulation, and decision-making. Comprising structures such as the prefrontal cortex (PFC), amygdala, hippocampus, and anterior cingulate cortex, and mediated by neurotransmitters like oxytocin, dopamine, and serotonin, the Social Brain serves as the neural substrate of social cognition [1–3].

As the complexity of social life increased throughout evolution, the Social Brain co-evolved to support behaviors essential for survival and species propagation [4,5]. In humans, its functions are not only crucial for interpersonal interactions but also for broader mental and physical health outcomes. Disruptions to this system whether through neuropsychiatric disorders, developmental anomalies, or environmental stressors can lead to profound dysfunctions in behavior, cognition, and somatic physiology [6].

Among the most significant environmental threats to the Social Brain is SI – the objective reduction or absence of social interaction [7]. While SI is a subtheme within this review, it provides a critical lens through which the vulnerability and adaptability of the Social Brain can be examined. SI acts as a chronic stressor and has been shown to affect the structure and function of the Social Brain, leading to emotional, cognitive, and physiological dysregulation [5,8]. Importantly, SI must be distinguished from loneliness, a subjective emotional experience, although the two often coexist [5].

This review begins by defining the architecture and functional domains of the Social Brain. We then explore how SI through its interruption of normative social signaling affects neural integrity and behavior across species. By drawing on both human and animal research, we aim to clarify the mechanisms by which the absence of social stimuli can alter the Social Brain and contribute to the development of neuropsychiatric conditions among which are anxiety, depression, addiction, schizophrenia, Alzheimer’s disease, and others [9]. Our goal is to provide an integrated framework that positions the Social Brain as a central node in the understanding of isolation-related pathologies and as a target for potential interventions.

## Discussion

To grasp the multifaceted effects of SI on human behavior and disease pathogenesis, it is helpful to understand the neural infrastructure that underlies the functional capacity of social interactions. Central to this is the concept of the Social Brain, a constellation of functional neural networks spread across multiple interconnected brain regions and neurochemical systems that have evolved together with the human capacity to socialize. While the psychological and behavioral consequences of isolation are well-documented, they are ultimately mediated by disruptions in specific brain areas. Exploring the structure and function of the Social Brain provides a mechanistic lens through which the widespread effects of isolation can be interpreted, from altered social cognition to stress-related pathology. In the following sections, we dissect the components of the Social Brain, their roles in normative social behavior, and the ways in which social deprivation compromises their integrity.

### The social brain

**Fig. 1:**
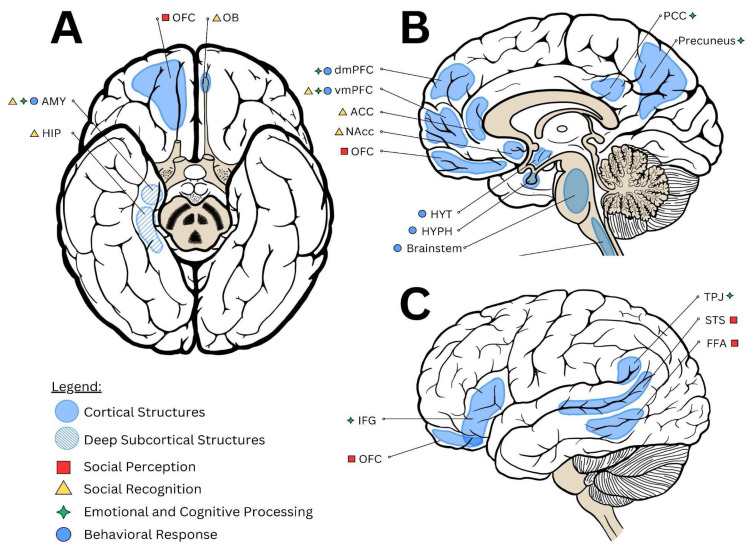
The neuroanatomical correlates of the Social Brain regions in a human brain; (**A**) Inferior view; (**B**) Medial view through sagittal section; (**C**) Lateral view; Structures are marked on the brain illustration with blue contour; Solid fill represents cortical structures; Lined fill represents deep subcortical structures; Each structure is labeled with a color-coded shape to represent associated function: Red square – social perception; Yellow triangle – social recognition; Green diamond – emotional and cognitive processing; Blue circle – behavioral response; ACC – Anterior Cingulate Cortex; AMY – Amygdala; FFA – Frontal Face Area; HIP – Hippocampus; HYPH – Hypophysis; HYT – Hypothalamus; IFG – Inferior Frontal Gyrus; NAcc – Nucleus Accumbens; OB – Olfactory Bulb; OFC – Orbitofronal Cortex; PCC – Posterior Cingulate Cortex; PFC – Prefrontal Cortex; STS - Superior Temporal Sulcus; TPJ – Temporoparietal Junction; dm – dorsomedial; vm – ventromedial. *Illustrations created by hand and labeled with the help of Canva.com*

The Social Brain encompasses a network of functionally interconnected regions including the medial prefrontal cortex (mPFC), temporo-parietal junction (TPJ), amygdala, hippocampus, superior temporal sulcus, and fusiform face area – that collectively govern social cognition, perception, emotional processing, and social behavior [10], are illustrated in Figure 1. These domains, which include mechanisms such as facial recognition, Theory of Mind (ToM), empathy, and behavioral output, rely on continuous social input for their development and functional integrity. SI, loneliness, and chronic stress impair these processes at multiple levels. For instance, SI has been shown to reduce gray matter volume in regions like the superior temporal sulcus and disrupt connectivity within the default mode network (DMN) a circuit tightly linked to introspective social cognitive processes such as mentalizing and emotional self-reflection [11,12]. Dysfunction in these circuits manifests in widespread impairments across psychiatric conditions, including major depressive disorder, schizophrenia, autism spectrum disorder, post-traumatic stress disorder, and attention-deficit/hyperactivity disorder, all of which show deficits in social cognition and DMN activity [13–16]. Moreover, animal models reveal that SI disrupts social recognition memory by altering hippocampal and prefrontal circuits, and impairs the encoding of social familiarity *via* the mPFC-nucleus accumbens pathway [17,18]. These findings underscore that the social brain is not merely a passive processor of social information, but a dynamic system whose structure and function are shaped and potentially damaged by the presence or absence of meaningful social interactions. When these mechanisms break down, the result is not just social dysfunction but increased vulnerability to psychiatric illness, maladaptive behavior, and altered neuroendocrine and autonomic outputs [7,8,19]. As such, the social brain is a central target of isolation-induced neuropathologies.

#### Social cognition and the social brain’s functional organization

Social cognition is an umbrella term that can be used to encompass the mental processes which mediate social interaction. Those can be broken down into several in(ter)dependent processes, which take place in specific structures within the Social Brain: social perception & recognition, emotional and cognitive processing, like mentalizing, and ToM, and the behavioral outcome [8].

Reportedly, social cognition is impaired to various degrees in many psychiatric and neurodegenerative disorders [20] including depression [21], generalized anxiety disorder, social phobia, and post-traumatic stress disorder [13], attention-deficit/hyperactivity disorder [14], addictions [22], schizophrenia [16], neurodegenerative diseases [23], and autism spectrum disorders [24,25].

#### Social perception, recognition and memory

Social perception is the process that describes the processing of visual and other sensory information and its identification as a conspecific social partner of the same species. In vision-dominant species such as primates, this is achieved primarily by evaluating information such as facial features [26] and eye gaze [27].

At the center of this process are the fusiform gyrus, specifically the fusiform face area [28] and the superior temporal sulcus [29]. Decreased grey matter in the left posterior superior temporal sulcus has been linked to deficient social perception among lonely individuals [29]. Additionally, specifically early face perception deficits have been observed among individuals with excessive internet use [30] a form of digital addiction, strongly linked to a spectrum of psychiatric disorders [31].

Social recognition is the process by which identified conspecifics of the same species are recognized as familiar ones. This occurs through retrieval of information from the social recognition memory, which is formed based on previous encounters with that individual [18]. Anatomical brain regions credited with generating social recognition memory include the anterior cingulate cortex, amygdala, and the hippocampus [18] as well as the Main and Accessory Olfactory systems [32], and mPFC [33].

Evidence from animal studies suggests that in mice for example, SI manifests as impaired social recognition – SI harms the encoding of social familiarity information by the mPFC-Nucleus Accumbens circuit [17], as well as decreases the tonic activity of glutamatergic signals between the hippocampus and the olfactory cortex [34], responsible for social recognition memory retrieval.

#### Cognitive and emotional processing

Mentalizing, empathy and ToM, while often used interchangeably, are largely overlapping but can be viewed as distinct processes, which provide the emotional and cognitive context to perceived social stimuli.

Mentalizing, or affective cognition, is a process which accounts for the attribution of mental and emotional states to oneself and others. Empathy, is the ability to share the emotional experience inferred from the other’s state, while ToM, or non-affective cognition, deals with higher metacognition and more complex mental reasoning such as beliefs, goals, desires, and intentions of one’s self and others [35–40]. Brain regions commonly viewed as the core of the mentalization network include the TPJ [41], posterior cingulate cortex/precuneus [42], mPFC [43,44] and the inferior frontal gyrus [45].

Schizophrenia [46], depression [47], attention-deficit/hyperactivity disorder [14] and autism spectrum disorder [48] are examples of disorders which show impaired mentalization. A study which analyzed brains of lonely people using multiple imaging modalities, reported observable changes in both grey matter and functional connectivity within the DMN [12], a collection of brain structures which largely overlaps with the Social Brain, which includes the mPFC, posterior cingulate cortex and TPJ, among others [11].

The DMN is active during passive behavior, when an individual’s attention is focused preferentially on internal cognitive concepts such as ToM, remembering past events, mentalizing and processing emotions of one’s self and of other individuals’, rather than the perception of external environment [15,49].

Changes in this network and in its connectivity with other neural networks have been implicated in psychosis, schizophrenia [50], depression, Alzheimer’s disease [11], autism spectrum disorder [51], post-traumatic stress disorder [52] and other psychiatric conditions, possibly as part of their pathogenesis. These regions may be of interest, when considering how SI and other social stressors affects the social brain.

#### Behavioral outcome

**Fig. 2:**
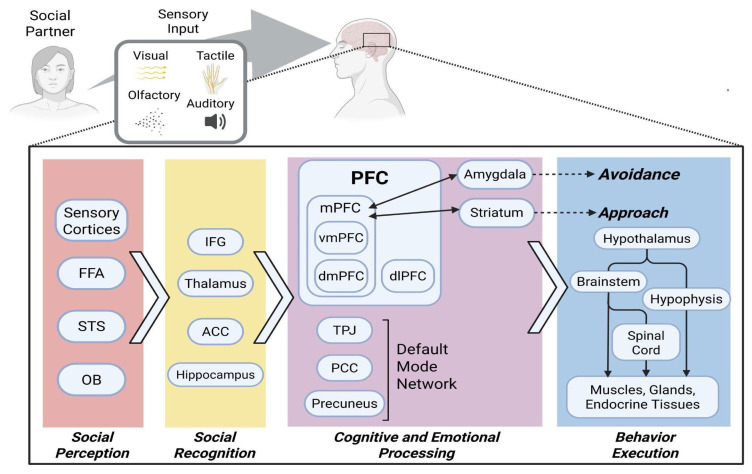
A hypothetical model depicting the neural processing of social stimuli; Processing begins with perception of a stimulus as a social signal and its recognition in context, this is followed by complex cognitive processing which involves activation of DMN structures and introspective thinking, with the PFC serving as the central hub for integrating this information with emotional content. PFC-Striatal connections favor approach while PFC-Amygdalar circuits favor avoidant behavior, which is likely the underlying mechanism of social withdrawal seen in psychiatric pathologies and isolation-induced conditions. Finally, the resulting behavioral response is executed using downstream pathways; Information is processed from left to right, each processing phase involves several structures; Boxes represent structures; Diamond-shaped boxes represent resulting behavior; Arrows demonstrate direct connections; Dashed arrows represent indirect influence; ACC – Anterior Cingulate Cortex; OB – Olfactory Bulb; DMN – Default Mode Network; FFA – Frontal Face Area; IFG – Inferior Frontal Gyrus; PCC – Posterior Cingulate Cortex; STS – Superior Temporal Sulcus; TPJ – Temporo-Parietal Junction; PFC – Prefrontal Cortex; vm – ventromedial; dm – dorsomedial; dl – dorsolateral. *Created with BioRender.com*

The behavioral outcome is the executive endpoint of cognitive processing. The PFC can be viewed as the central hub for processing endogenous state and exogenous stimuli, through bilateral circuits with the amygdala and striatum among many other brain areas (according to the triadic nodes hypothesis, as reviewed in greater detail by [53]). It then gives off efferent projections which manifest as the behavioral response (Fig. 2). The outcome is in both voluntary (somatic movement) and involuntary (facial micro-expressions) motor actions, as well as visceral neurohumoral reactions (humoral signalization, neuronal signal transduction), all mediated by the motor cortices, brainstem, hypothalamic-pituitary-adrenocortical (HPA) axis and motor and autonomic descending spinal tracts [19].

It must be mentioned, that the discussed processes cannot be attributed to single neuron groups or cell bodies, but rather a network of interdependent regions which often have multiple functions, for example as is the case of the fusiform face area and face perception [54].

The functional integrity of the Social Brain is critically dependent on consistent social input. Social experiences shape the development and plasticity of this network throughout life, while deprivation of such input as seen in conditions of SI can induce widespread disruptions in its structure and function [7,55]. This disruption extends beyond cognitive or emotional domains; SI elicits measurable changes in social perception, recognition, affective processing, and executive behavior, all of which are mediated by core regions of the Social Brain, including the mPFC, amygdala, hippocampus, and TPJ [12,17]. Converging evidence from both human and animal studies shows that chronic SI can dysregulate DMN connectivity, impair social cognition, and increase vulnerability to psychiatric and neurodegenerative disorders [11,34].

Thus, the social brain not only encodes the mechanisms of social behavior – it also constitutes a primary target of isolation-induced stress. In the sections that follow, we synthesize behavioral and biological findings from both animal and human studies to elucidate how SI disrupts this core social circuitry, resulting in profound implications for health and disease.

### Social isolation

The functional integrity of the Social Brain relies on regular interpersonal interactions, and its disruption through SI poses a substantial risk to health and well-being. In humans, SI is associated with dysregulation of neural circuits involved in social cognition, elevated risk for psychiatric and neurodegenerative disorders, and increased morbidity and mortality [7,12,56]. Understanding the specific consequences of SI in humans is therefore essential not only for characterizing how socially relevant neural networks are shaped by isolation, but also for informing translational efforts grounded in both human and animal research.

### Disruption of social behavior in humans and animals

Beyond anxiety and depression, SI profoundly disrupts social behaviors themselves, altering how individuals engage with their peers, and this is evident across a variety of species. In rodents, prolonged SI reduces social exploration and preference, evidenced by diminished time spent investigating novel conspecifics in the three-chamber social interaction test [57]. Isolated mice also show heightened aggression [58,59] and abnormal social communication patterns, such as altered ultrasonic vocalizations [60].

In non-human primates, SI particularly in early-life leads to social withdrawal and stereotypical behaviors, which persist into adulthood together with inappropriate social interactions [61], but in certain cases may be reversible [62]. Such alterations are evidenced with elevated cortisol, and may partially be explained by disrupted hippocampal neurogenesis [63], decreased positive social interactions such as play and sex, increased fearfulness, and more aggressive behavior as well as bizarre repetitive movements [64].

Importantly, human studies mirror these patterns: socially isolated individuals display reduced prosocial behavior, diminished trust, and impaired ToM performance, reflecting a breakdown in social attunement [12,65]. The impairment of social behavior under SI is thus not merely a byproduct of mood disorders, it reflects a core alteration in the neural circuits governing social interaction, highlighting the need for interventions that address social cognitive deficits alongside emotional symptoms.

### Social isolation in humans

**Fig. 3:**
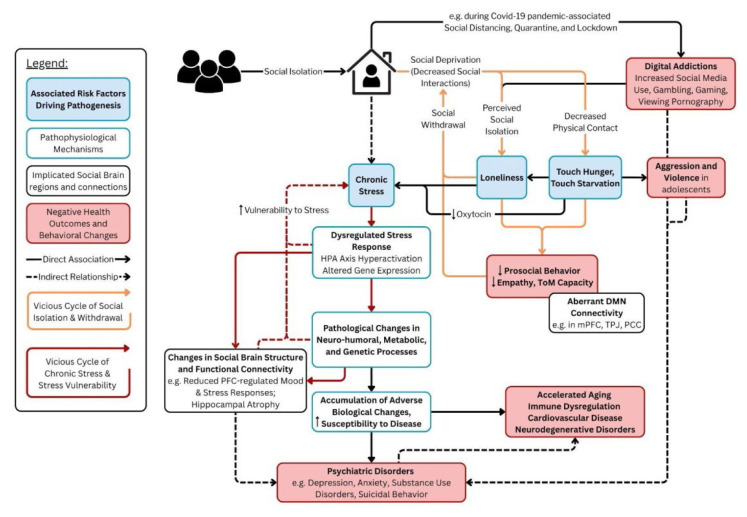
Social Isolation as Chronic Stress in Humans: A suggestive model demonstrating the HPA axis hyperactivation as the centeral pathophysiological processes (white boxes with blue borders) of chronic stress that stems from prolonged social isolation, potential risk factors (blue boxes) possibly contributing to the development of isolation-induced disorders, and regions of the Social Brain (white boxes with black borders) that are thought to play an important role. The end-points (red boxes) are represented by adverse behavioral alterations and disease pathogenesis; The vicious cycles that are thought to drive this pathogenesis are Chronic Stress and related changes (red arrows) and Social Isolation followed by Loneliness and Social Withdrawal (yellow arrows), which reinforces the Chronic Stress cycle. Arrows represent the direction of the relationship; straight arrows indicate likely direct associations; dashed arrows represent an indirect relationship; HPA – Hypothalamic Pituitary Adrenocortical axis; DMN – Default Mode Network; PFC – Prefrontal Cortex; mPFC – medial Prefrontal Cortex; TPJ – Temporoparietal Junction; PCC – posterior Cingulate Cortex. *Created with BioRender.com*

SI can be viewed as the objective state in which an individual is separated from their society and community with the decreased number and/or quality of interactions. As a result, socially isolated individuals may experience the adverse consequences of isolation such as loneliness, decreased physical contact and touch, decreased social interactions, and suffer from chronic stress precipitated by these factors [5,56]. The following chapters discuss how these factors are individually related to morbidity, and explain what likely stands behind the negative health consequences of SI. A flowchart describing the mechanisms by which SI impacts human wellbeing, and the relationship between these factors, with chronic stress as the core driver of pathogenesis are illustrated in Figure 3.

#### Effects of social isolation on human social behavior and social cognition

SI profoundly disrupts social behavior and social cognition in humans. Socially isolated individuals tend to withdraw from social activities, reduce their frequency of interpersonal interactions, and show diminished prosocial behaviors, such as helping or cooperating with others [7]. Even perceived SI, provoked by active social exclusion, results in decreased prosocial behavior [66]. Importantly, SI impairs key cognitive processes involved in navigating social environments, including emotion recognition, empathy, and ToM capacity [67]. Neuroimaging studies reveal that socially isolated individuals show altered activation and connectivity within core social brain networks, including the mPFC, TPJ, and posterior cingulate cortex, regions critical for processing social information and evaluating trustworthiness [12,68]. As a result, SI not only reduces social engagement but also degrades the cognitive capacities that support adaptive, meaningful social interactions, reinforcing a vicious cycle of withdrawal and further isolation.

#### Mental health consequences of social isolation

SI is a well-established risk factor for a range of psychiatric conditions, including depression, anxiety disorders, substance use disorders, and suicidal behaviors. Longitudinal analyses across age groups have shown that individuals with persistent SI are at significantly higher risk of developing major depressive disorder and generalized anxiety disorder, independent of other demographic or health factors [69]. Importantly, SI and its perception are not only correlates of mental illness but can act as a risk factor: evidence suggests that lack of social connection disrupts affect regulation, increases negative cognitive biases, and impairs stress-buffering mechanisms, likely contributing to the development of depression [65].

Neurobiological studies reveal that SI enhances activity in brain regions associated with social pain, such as the anterior cingulate cortex, and increases amygdala reactivity, and amplifies emotional sensitivity to threats [70]. Furthermore, chronic SI elevates HPA axis activation, leading to sustained cortisol release, which over time contributes to hippocampal atrophy and reduced prefrontal regulation of mood and stress responses [71].

Meta-analyses confirm that loneliness, which is often intertwined with SI, significantly predicts depression, with effect sizes comparable to other psychosocial risk factors [72]. Moreover, a systematic review found that both objective SI and subjective loneliness are robust predictors of suicidal ideation and suicide attempts across populations [73]. Prolonged SI may lead to loneliness, boredom and decreased physical activity which when sustained for a prolonged period may advance chronic stress and adversely affect mental and physical health [74]. Notably, SI is a potent stressor that contributes to drug-seeking behavior and relapse vulnerability that is often overlooked in addiction research [75].

#### Touch deprivation in social isolation

Another stressor that can be caused by SI and can be a major contributor to chronic stress precipitated by it, is touch deprivation. Tactile sensations are known to play an important role in social interaction already from the early stages of development [76] and not only in humans [77]. Touching other individuals is integral to interpersonal connection and is a fundamental part of human and non-human communication, which can deeply and immediately affect the emotional, psychological, and physical state of those involved [76,78].

Already from infancy, touching plays an important part not only in learning but also in the development of the social brain [79,80], while neglect in positive affective touch in children may negatively impact behavioral, psychological, emotional, and physical development [81,82].

Touch results in a release of oxytocin, a key hormone involved in social bonding, affection and attraction, trust, and a range of positive emotions, especially when there is a preexisting positive relationship towards the toucher’s identity [78,83]. The positive effects of touch are even used intentionally by medical personnel to improve patient healing and reduce anxiety [84] and it has been shown that oxytocin, released following positive affective touch, decreases HPA activation, blood pressure and cortisol plasma levels [85–87], evidently decreasing stress levels.

As could be expected, when humans are isolated, they are not only deprived of the positive effects of social/affective touch (as well as sexual touch, if they live alone), but are also adversely affected by its absence, developing what can be termed ‘touch hunger’ or ‘touch starvation’ [88], increasing stress, loneliness, depression, and anxiety, and in adolescents, even fueling aggression and violence [89].

#### Public health relevance

In addition to SI’s established effects on increased physical and mental morbidity and mortality [90], other aspects of the effects of SI on humans have been widely observed with the introduction of mandatory quarantine and social distancing measures in public spaces used to combat the COVID-19 pandemic owing to it being considered an effective tool to mitigate infection transmission rates between individuals, commonly suggested by various health agencies such as the US Center for Disease Control and Prevention [91] and the World Health Organization [92]. Both loneliness and SI have been highly prevalent even before COVID-19 and sometimes referred to as a “behavioral epidemic”, but these numbers are progressively worsening [93].

While considered an effective measure in dealing with infection rates, social distancing can however by increasing chronic stress, impair immunity thus directly increasing infection likelihood [94]. Quarantine, a public measure where isolation is enforced by repercussions, has been linked to increased development of psychological distress such as anxiety and depression, compared to populations unaffected by quarantine [95], however, the literature is conflicted, and contradicting studies have also been published regarding the role of quarantine itself, and other factors such as socioeconomic status may carry more weight, in the consequential negative impact of quarantine on psychological state [96].

Behavioral changes induced by loneliness, isolation, and chronic stress are also apparent. During the COVID-19 pandemic, there were increased reports of substance abuse, relapse from abstinence, digital addictions and higher use of social media [97]. A narrative review on the subject of problematic use of the internet has identified online gaming, gambling and viewing pornography as factors potentially detrimental to mental health [98]. Moreover, those associations appear to be bidirectional, e.g. young adults with higher social media use tend to be 3 times more susceptible to loneliness [99].

### Social isolation in animals

While research in humans has provided valuable insights into the psychological, cognitive, and physiological consequences of SI, many mechanistic questions remain unresolved due to methodological and ethical constraints as well as the largely correlational nature of human studies – limiting the ability to dissect causal pathways or conduct experimental manipulations. This gap is effectively addressed by animal models, which offer powerful tools for probing the neurobiological, behavioral, and systemic effects of SI under tightly controlled conditions. Importantly, many of the neural circuits and stress pathways implicated in social behavior are highly conserved across mammalian species, e.g. findings from rodents and non-human primates, are particularly relevant for translational research [100,101]. These models allow researchers to manipulate the timing, duration, and social context of isolation, shedding light on how specific aspects of social deprivation shape neural development, stress responsivity, and social behavior [100,102]. By integrating human and animal research, we can develop a more comprehensive understanding of how SI disrupts core brain systems, social cognition, and affects regulation. The following sections synthesize evidence from animal models to illuminate the specific behavioral outcomes, social deficits, and biological mechanisms that underlie isolation-induced dysfunction.

#### Effects on social interaction and behavior

Social behavior is profoundly affected by SI in animals. Among the observable alterations in behavior: isolated rodents show reduced social preference in the three-chamber social interaction test, demonstrating lower motivation for social contact [57]. Juvenile isolation impairs adult social recognition and affiliative behavior, indicating long-lasting developmental consequences [103]. In addition, SI alters mating behavior, maternal care, and play behavior, all of which depend on intact social motivational systems [104]. These findings suggest that the absence of social interaction not only affects emotional regulation but also degrades the capacity to engage in meaningful social exchanges, with implications for social competence and group cohesion.

#### Social isolation induces anxiety-like and depression-like behavior

Animal models have consistently shown that SI induces robust anxiety- and depression-like behaviors across species, and these have been observed across a battery of validated behavioral tests. In rodents, prolonged SI increases immobility in the forced swim test and tail suspension test, both classic markers of behavioral despair [100]. Isolated animals also show reduced sucrose preference, indicating anhedonia, and heightened anxiety-like responses in the elevated plus maze and open field test [102]. The lack of social touch has also been underscored as a contributor to the development of depressive/anxious phenotype in mice [105]. Notably, prairie voles separated from bonded partners exhibit increased passive coping behaviors together with physiological markers of stress, such as elevated corticosterone, paralleling human patterns of social stress [106].

Additionally, observable depressive/anxious changes in behavior, compared to grouped-housed mice, socially isolated mice display a higher degree of locomotor activity in general, increased duration of immobility in the tail suspension test, less time spent in the center of the open field test, [107] which correlates with higher degree of depression-like behavior. Socially isolated rats display an impaired prepulse inhibition response as well as an abnormal behavioral response when introduced into novel environments, characterized by locomotor hyperactivity, and prolonged habituation [108,109]. These behavioral phenotypes mirror certain key features of human affective disorders, reinforcing the translational relevance of SI animal models.

#### Biological mechanisms of social isolation stress

At the biological level, SI triggers profound neurochemical, structural, and endocrine changes, which often coincide with the development of depressive-/anxious-like behavioral phenotype. Isolated animals show altered dopamine and serotonin signaling in the PFC and hippocampus, key regions regulating reward processing and emotional balance [110]. SI also decreases hippocampal neurogenesis, disrupts synaptic plasticity, and increases expression of stress-responsive genes [107].

SI can be viewed as a chronic extrinsic stressor. Chronic stress, in general, can arise from different causes, but there are many similarities in the resulting consequences regardless of its etiology, many of which are shared across species. Chronic stress can be defined through the biological lens as chronically elevated glucocorticoid levels, which in case of external stressors is precipitated by hyperactivation of the HPA axis [111]. Chronic stress is a risk factor for neuropsychiatric disorders, and has been associated with changes in the brain such as decreased expression of several neuroplasticity-related genes in the hippocampus and PFC, concomitant with the development of depressive-like behavior [107]. Endocrinologically, SI elevates HPA axis activity, resulting in chronically elevated glucocorticoids, which over time impair immune function and increase oxidative stress [112]. These biological mechanisms not only mediate the behavioral effects of SI but also contribute to its long-term impact on health, including increased vulnerability to metabolic, cardiovascular, and neurodegenerative disorders.

**Fig. 4:**
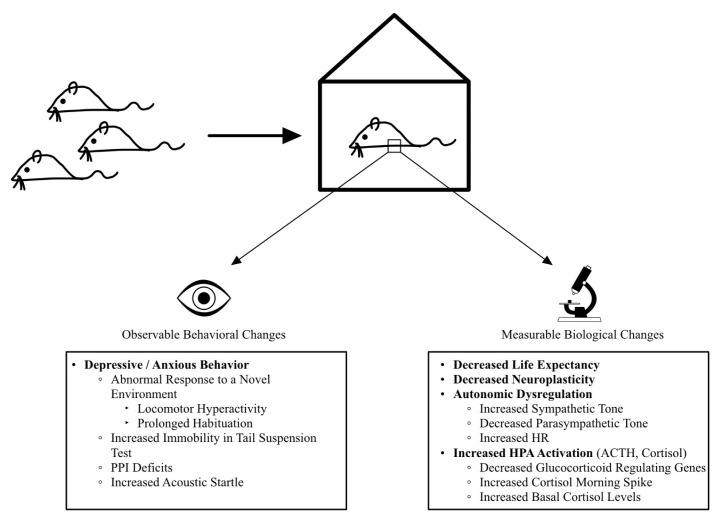
Behavioral (left) and Biological (right) changes observed in socially isolated non-human mammals, especially rodents, tend to occur together and are consistently replicated across studies; Abbreviations: PPI – Prepulse inhibition; HR – Heart rate; HPA – Hypothalamic-Pituitary-Adrenocortical axis; ACTH – Adrenocorticotropic Hormone.

The effects of SI on study subjects can be described using behavioral and biological perspectives (Fig. 4). There are firm ties between the two, but many of them remain to be elucidated. The effects of the biological consequences of SI are numerous and complex, and are beyond the scope of this paper. However, there is overwhelming evidence underscoring the significance of SI’s effect on biological processes, and since there is a close relationship between biology and behavior which contributes to the understanding of behavioral alterations some examples deserve to be mentioned.

SI has been associated with numerous objective adversities, which are comparably consistent across species: decreased life expectancy in invertebrates [113]; increased sympatho-adrenomedullary activation in in response to stress, increased obesity and type 2 diabetes mellitus, and delays in the positive effects of running on adult neurogenesis in rodents [114–116]; increased morning spikes in cortisol, decreased expression of genes regulating glucocorticoid response in the frontal cortex, increased basal cortisol concentrations, and decreased lymphocyte proliferation in response to mitogens, increased oxidative stress in the aortic arch of mammals [112,117–120]. In prairie voles, SI from bonding partners produces increased depressive behavior and autonomic imbalance characterized by an increased sympathetic tone and decreased parasympathetic tone. Concomitantly, increased heart rate, heart rhythm dysregulation, and increased levels of adrenocorticotropic hormone and cortisol have been measured [121]. Prepulse inhibition deficits have also been observed in SI-reared mice using the acoustic startle test concomitant with decreased dopamine and acetylcholine levels in the PFC [122]. These findings show quantifiable changes in signaling systems that are involved in social functions and are highly preserved across species, including humans, offering potential directions for future exploration.

## Conclusions

Social interactions require a complex neural infrastructure spread out across brain regions and neurohumoral systems, which can be referred to as the Social Brain. It is highly conserved across mammalian species throughout evolution, but is particularly developed in humans. The Social Brain provides the functional capacity for the different aspects of social cognition, like social perception, emotional processing, and the efferent connections that carry important signals which determine both conscious behavior and unconscious physiological responses to social stimuli. Therefore, when an individual is submitted to social isolation, it is this framework that harbors the target structures, connectivity networks, and social cognitive functions that are often adversely impacted by isolation. Those structures and functions largely overlap with many of the ones that are affected in neuropsychiatric disorders, suggesting that social isolation and the resulting chronic stress are major pathogenesis drivers of those conditions.

Social isolation by way of the consequent deprivation from social interactions acts as a chronic stressor, and there is robust evidence from human studies that it is a significant risk factor which contributes to an individual’s vulnerability to disease, propagates vicious cycles of increased stress reactivity and chronic stress, negatively affects social behavior which reinforces social withdrawal, results in adverse mental health outcomes, and ultimately precipitates morbidity, particularly neuropsychiatric disorders.

This evidence from humans is largely mirrored in both behavioral and biological animal research. There is significant overlap between the behavioral changes observed in isolated animals and those that serve as animal models of human neuropsychiatric disorders, like locomotor hyperactivity and prolonged habituation to novel environments, e.g. in rodent models of depression. The underlying biological alterations observed concurrently with the behavioral changes offer additional strong evidence that often correlates with comparable changes in humans, such as HPA axis hyperactivation and its subsequent detrimental effects.

Many limitations of this work persist, like the translatability of animal research to humans, difficulties in simplifying complex neural functions and systems, and the challenging task of synthesizing a solid hypothesis from the largely correlative nature of human studies in psychiatry. The neurotransmitter systems that facilitate the Social Brain’s functions have largely been omitted from this manuscript for simplicity purposes and are beyond the scope of this review, however, investigating the relationship between these systems will improve the model suggested in this article. Future research should explore how protective factors like touch, social engagement and community inclusion may buffer against the detrimental effects of isolation or even counter pathogenic processes increasing human resilience. Incorporating longitudinal multimodal testing to understand how brain structure and connectivity are influenced by isolation would shed light on neurohumoral processes within the social brain, offering specific intervention targets for prevention and therapy. Animal studies need to integrate both isolated and grouped housing, and include a re-socialization period to evaluate how social isolation acts as an effect modifier of pathogenesis and whether these effects are reversible. Testing how sex differences affect behavioral changes in both humans and animals may reveal additional mechanisms and increase the research translatability.

The rising prevalence of conditions like depression and neurocognitive disorders, together with the increasing psychosocial impact of loneliness and social withdrawal on public mental health suggest that studying social isolation and the underlying neurobiological architecture may reveal modifiable risk factors, provide a deeper understanding of human brain pathologies, and offer actionable targets for novel therapeutic approaches to some of the most burdensome diseases that plague modern human society.
